# The Surgery of the Kidney

**Published:** 1886-12

**Authors:** 


*The Surgery of the Kidney*. By W. BRUCE CLARKE, M.A., F.R.C.S. pp. 176. London: H. K. LEWIS.

This work, coming after Mr. Morris's admirable monograph, does not appear at the most favourable time. It is the Jacksonian Prize Essay of 1886, and was written before Mr. Morris's work appeared. What the Jacksonian Prize is, and how, why, and wherefore it is awarded, we have not the remotest idea. But if, in 1886, it was intended to be given to the author of the best work on renal surgery, then, without a doubt, Henry Morris ought to have received it.

Compared with Morris's work, this one is sketchy and superficial. But it is readable and trustworthy. In these bustling times, when the greatest amount of practical show is sought from the least possible effort of the mind, a work like that of Mr. Bruce Clarke is sure to be appreciated. About details we need not be hypercritical. If we hurriedly reach down our English Dictionary when we meet such a word as "mortary," and wonder whether an Oxford graduate *can* make up his mind as to the spelling of nephroraphy, or nephrorhaphy, or nephrorraphy, or nephrorrhaphy (he wisely gives a choice), we cannot fail to be struck with the easy, almost precocious, definiteness with which the author handles theories and lays down laws of practice. As compared with the elaborate caution in Mr. Morris's monograph, the unhesitating confidence in this one of Mr. Bruce Clarke's is refreshing and even stimulating. Saying the best of both works, Morris's book might be spoken of as able and profound, while Clarke's is clever and taking. Saying the worst, it would be scarcely wrong to speak of the former as heavy, of the latter as flighty.

We have no space for examples; but we would call attention to the last chapter, on "Methods by which one ureter can be temporarily occluded," as being a fair specimen of the whole work. It is clear the author has no experience of what he writes about; indeed, he makes pretensions to none. Yet he is able to write a clear, facile, and generally condemnatory critique of the work of every one else. "Catheterism of the ureters" (he says in the concluding paragraph) "is destined possibly to aid in the diagnosis of a stone in the ureter or in the pelvis of the kidney; but it is difficult to see how it could give any clear evidence of the secreting power of a kidney unless the catheter could be securely fixed in the ureter for some hours at the very least." Now, if catheterism of the ureter was intended only to give evidence on the "secreting power" of a kidney, and if the catheter were once passed, surely there could be no difficulty in leaving it in for a few hours. He does not tell us that secreting power can be deduced from other observations, and that the characters of the urine can be detected from a drachm as easily as from a pint. Again: the casual manner in which he speaks of sounding a stone in the pelvis of the kidney through the ureter is to a practical mind somewhat irritating. It savours of the free and easy treatment which precocious children give to sacred and profound subjects. The original and enthusiastic surgeon may see and feel with the author; the plodding seeker after information will be left bewildered and amazed.

There is a long bibliography, which is in general harmony with the character of the book. It is neither complete nor representative. Take "movable kidney," for instance. We might pardon the omission of such names as those of Martineau, Rigal, Callais, Buret, or Newman; but no excuse whatever can be made for the neglect of Landau. The same criticism might be applied to every other subject. In almost every instance the author seems to have failed to note the best work of the ablest men. He has picked up and digested such crumbs as fell in his way; he has not groped about for the strongest pabulum. We heartily admit that the affectation of bibliographical knowledge is generally a nuisance; but when, in the preface, our author apologises for an abundance of references which is a poverty, and a completeness of bibliography which he hopes may "lessen the labours of those who are pursuing a similar line of inquiry," we deem it necessary to speak out our candid opinion. And not only are the abundances not complete nor representative; they are sometimes not even correct in quotation of statement.

We have said that the work is pleasant, readable, clever, and mostly trustworthy. The ignorant and honest surgeon will get little guiding information; the critical and informed man will find much to please and interest him. But renal surgery will not be advanced by the book. It is an abbreviated epitome of opinions such as the writer has come across; it is by no means a full or authoritative exposition of knowledge. If it is the Jacksonian Prize Essay, we hope it is far below the standard generally attained by that production.

